# Genome-Wide Analysis Identified a Set of Conserved lncRNAs Associated with Domestication-Related Traits in Rice

**DOI:** 10.3390/ijms22094742

**Published:** 2021-04-29

**Authors:** Huang He, Yan-Fei Zhou, Yu-Wei Yang, Zhi Zhang, Meng-Qi Lei, Yan-Zhao Feng, Yu-Chan Zhang, Yue-Qin Chen, Jian-Ping Lian, Yang Yu

**Affiliations:** 1 Guangdong Provincial Key Laboratory of Plant Resources, State Key Laboratory for Biocontrol, School of Life Science, Sun Yat-Sen University, Guangzhou 510275, China; hehuang_@163.com (H.H.); zhouyf65@mail.sysu.edu.cn (Y.-F.Z.); 18947169884@163.com (Y.-W.Y.); zhangzh295@mail2.sysu.edu.cn (Z.Z.); leimq@mail2.sysu.edu.cn (M.-Q.L.); fyz729@163.com (Y.-Z.F.); zhyuchan@mail.sysu.edu.cn (Y.-C.Z.); lsscyq@mail.sysu.edu.cnv (Y.-Q.C.); 2 Guangdong Provincial Key Laboratory for Crop Germplasm Resources Preservation and Utilization, Agro-Biological Gene Research Center, Guangdong Academy of Agricultural Sciences, Guangzhou 510640, China

**Keywords:** agronomic traits, conservation, crop domestication, cultivated rice, lncRNAs, wild rice

## Abstract

Crop domestication, which gives rise to a number of desirable agronomic traits, represents a typical model system of plant evolution. Numerous genomic evidence has proven that noncoding RNAs such as microRNAs and phasiRNAs, as well as protein-coding genes, are selected during crop domestication. However, limited data shows plant long noncoding RNAs (lncRNAs) are also involved in this biological process. In this study, we performed strand-specific RNA sequencing of cultivated rice *Oryza sativa* ssp. *japonica* and *O. sativa* ssp. *indica*, and their wild progenitor *O. rufipogon*. We identified a total of 8528 lncRNAs, including 4072 lncRNAs in *O. rufipogon*, 2091 lncRNAs in *japonica* rice, and 2365 lncRNAs in *indica* rice. The lncRNAs expressed in wild rice were revealed to be shorter in length and had fewer exon numbers when compared with lncRNAs from cultivated rice. We also identified a number of conserved lncRNAs in the wild and cultivated rice. The functional study demonstrated that several of these conserved lncRNAs are associated with domestication-related traits in rice. Our findings revealed the feature and conservation of lncRNAs during rice domestication and will further promote functional studies of lncRNAs in rice.

## 1. Introduction

The *Oryza* genus is an ideal model for studying mechanistic insights into crop domestication. The Asian cultivated rice *Oryza sativa* is one of the most important staple crops worldwide and was domesticated from its wild progenitor *Oryza rufipogon* ~10,000 years ago [1]. As cultivating high-yield rice varieties is the core objective in ancient breeding, a number of morphological and physiological changes appeared during the domestication process, especially the yield-related traits including panicle complexity and grain size [1,2,3,4]. For example, *O. rufipogon* and *O. sativa* exhibit strikingly different panicle architectures, from a low branching complexity and small grain size in *O. rufipogon* to a more complex panicle and larger seeds in *O. sativa* [5,6,7]. A similar divergence of domestication-related traits was also observed between the African rice *O. glaberrima* and its wild ancestor *O. barthii* [8].

Previous studies have reported that the two Asian rice varieties, *O. sativa* ssp. *japonica* and *O. sativa* ssp. *indica*, were domesticated from different *O. rufipogon* populations by two independent domestication events [9]. With the rapid advance of genome or transcriptome sequencing technologies, considerable genetic diversity have been characterized from wild and cultivated rice [10]. Pan-genome analysis of the whole set of coding genes among 66 rice accessions revealed extensive genomic variation in cultivated and wild rice. Functional analyses of gene variations that are targeted by artificial selection also provided crucial insights into rice domestication [11]. A couple of genes have been evidenced to be selected, such as the grain size-related genes *GS3* and *SW5*, the *Ideal Plant Architecture 1* (*IPA1*), the panicle architecture-associated genes *LAX1* and *DEP1*, the tillering number- or angle-determining genes *MOC1*, *DLT* and *PROG1* and shattering-regulating genes *Sh4* and *qSH1* [5,7,12].

In addition to protein-coding genes, noncoding RNAs are found to be crucial regulators of rice domesticated traits as well. Several small RNAs, such as microRNAs miR164, miR390, miR395, and miR2118-triggered phasiRNAs are proved to be directly selected during rice domestication [13,14,15]. One nucleotide substitution in the miR156 target site of the *IPA1* gene disrupts miR156-mediated mRNA cleavage, leading to enhanced *IPA1* expression in panicles and conferred the ideal plant architecture for rice [16,17]. These observations suggested that small noncoding RNAs might play important roles in the domestication of rice. Except for the small RNAs, long noncoding RNAs (lncRNAs), ranged more than 200 nt in length, have also been reported to perform various functions in plant reproduction and defense [18,19,20]. A recent study characterized 3363 lncRNA transcripts from *O. rufipogon* and *japonica* rice cultivars, with 311 of them significantly downregulated in *japonica* compared to wild rice [21]. Following data analyses and transgenic experiments it was revealed that selection on these lncRNAs loci may be associated with increased starch content and grain weight [21]. Despite the fact that a subset of lncRNAs has been identified from *Oryza* species [18,19,20,21], there remains a significant number of yet undiscovered lncRNAs that need to be characterized from a comprehensive analysis of the combined *O. rufipogon–japonica-indica* transcriptomes.

In plants, a sequence conservation analysis of lncRNAs in five dicot and five monocot species suggested that high sequence conservation occurred in the majority of lncRNAs at the intra-species and sub-species levels, while the lncRNA sequences were highly diverged at the inter-species level [22]. These results provided knowledge to facilitate research of lncRNA function in evolution, however, no evidence has shown the comparison lncRNA sequence during domestication. As comprehensive morphological and physiological changes appeared during rice domestication, we hypothesized that lncRNAs might also be involved in this process. To clarify the characteristics of lncRNAs and their biological roles for agronomic traits changes during crop domestication, we performed a genome-wide analysis of lncRNAs from the seedlings and panicles of cultivated rice *O. sativa* ssp. *japonica* and *O. sativa* ssp. *indica* and the wild rice *O. rufipogon.* A number of lncRNAs expressed in wild rice were revealed to be shorter in length and have fewer exon numbers when compared with lncRNAs from cultivated rice. Comparative analysis and functional study demonstrated that many conserved lncRNAs are associated with agronomic traits in rice. Our results revealed the characteristics and conservation of lncRNAs during rice domestication and demonstrated the potential to improve plant agronomic traits by manipulating the expression of lncRNAs.

## 2. Results

### 2.1. Transcriptome-Wide Analysis of lncRNAs in Wild Rice and Cultivated Rice

To show vivid changes of the agronomic traits during rice domestication, we investigated the panicle architectures, seed-setting rate, and grain shape of three *Oryza* species: *O. rufipogon* collected from Hainan province, *O. sativa* ssp. *japonica* Nipponbare, and *O. sativa* ssp. *indica* 93-11. We found a series of profound changes appeared during domestication, including increased seed-setting rate, grain size, and grain weight, as well as a degenerated awn, hull color, and pericarp color (Supplementary Figure S1). Besides this, studies over the past decades have provided important information on protein-coding genes in regulating domestication traits, such as shattering, plant architecture, heading date, tiller angle, stress adaptation, and grain quality. In this study, we asked whether long noncoding RNAs could also be involved in the rice domestication process.

**Figure 1:**
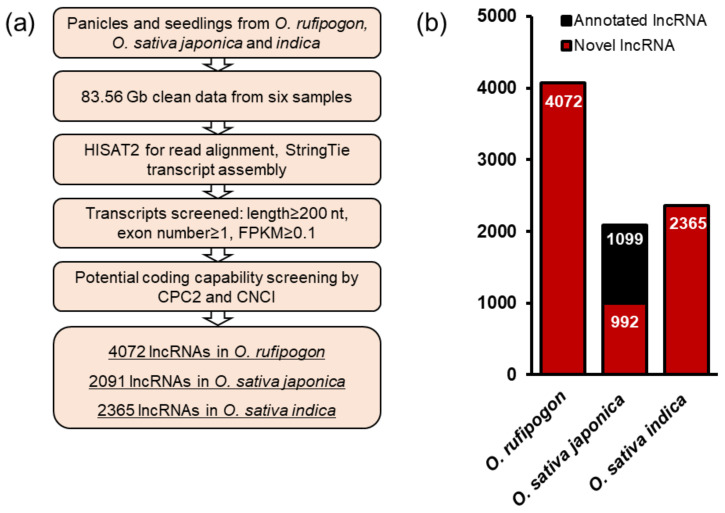
Identification of lncRNAs from wild and cultivated rice. (**a**) Pipeline used to identify lncRNA transcripts in this study. (**b**) Comparison of lncRNAs identified in this study from previously annotated rice lncRNAs. The red box represents the number of novel lncRNAs identified in this study, and the black box indicates the number of previously annotated lncRNAs in the literature [21,28] that are included in our datasets.

To answer this question, we first collected samples of panicles and seedlings from the wild rice *Oryza rufipogon,* and two cultivated rice, *O. sativa* ssp. *japonica* and *indica*. We performed strand-specific paired-end deep sequencing to characterize lncRNAs and obtained 83.56 Gb clean reads in these six datasets with 89.18% of them able to be mapped to the referenced genomes of wild and cultivated rice [23,24,25,26,27] (Table 1). An average of 76.64% of these reads from six datasets were uniquely mapped to the *Oryza* genomes, followed by transcript assembly using the StringTie tool and obtained a total of 45,579 (including 26,657 mRNAs), 40,097 (including 31,313 mRNAs), and 41,149 (including 28,686 mRNAs) transcripts in *O. rufipogon*, *japonica* and *indica* groups, respectively. Basic filter processes and potential coding capability screening were than performed to exclude protein-coding genes, small noncoding RNAs that were shorter than 200 nt, and transcripts with FPKM (fragment per kilobase of transcript per million mapped reads) less than 0.1 (Figure 1a). We finally obtained 8528 lncRNAs from the six datasets, including 4072 lncRNAs in *O. rufipogon*, 2091 lncRNAs in *japonica* and 2365 lncRNAs in *indica* (Figure 1b, Supplementary Tables S1–S3). In order to estimate whether these lncRNAs are novel or not, we aligned them with annotated lncRNAs in the reference genome database and previous literatures [21,28] and found more than a half of lncRNAs from *japonica* (1099 out of 2091, 52.56%) were known (Figure 1b and Supplementary Table S2), while none of the 4072 wild rice-derived lncRNAs and 2365 *indica* rice-derived lncRNAs were annotated before (Figure 1b, Supplementary Tables S1 and S3).

### 2.2. Characteristics Comparison of lncRNAs in Wild and Cultivated Rice

**Figure 2:**
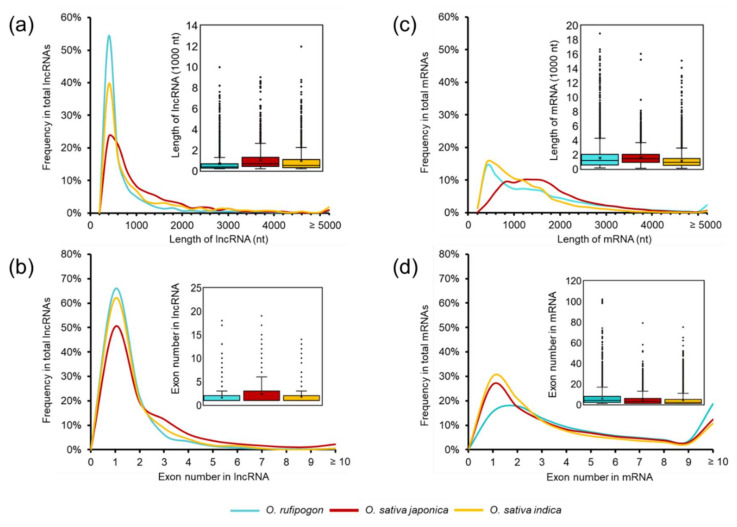
Frequency distribution comparisons of lncRNAs and mRNAs between wild and cultivated rice. (**a**) The length of lncRNAs. (**b**) The exon number of lncRNAs. (**c**) The length of mRNAs. (**d**) The exon number of mRNAs.

Since most of the lncRNAs identified in this study were novel, we asked whether they had distinctive features between wild and cultivated rice. We performed *t* statistics for the following comparison between wild and cultivated rice and found lncRNAs in wild rice appeared to be shorter than that of cultivated rice, with the median length of 370 nt for wild rice-derived lncRNAs, while the median length of lncRNAs in *japonica* and *indica* rice were 670 nt (*p* < 0.0001) and 508 nt (*p* < 0.0001), respectively (Figure 2a). The wild rice-derived lncRNAs had fewer exons than that of cultivated rice, with 1.61 exons on average in wild rice versus 2.37 (*p* < 0.0001) and 1.76 (*p* < 0.0001) exons in *japonica* and *indica*, respectively. The percentage of single-exon lncRNAs in wild rice is 65.83%, and the percentage in *japonica* and *indica* rice were 50.39% and 61.96%, respectively (Figure 2b). We also explored the characteristics of mRNAs in our datasets. Distinct from lncRNAs, the median mRNA length of protein-coding genes in wild rice was 1178 nt, which was lower than in japonica rice (1425 nt, *p* < 0.0001), but higher than in indica rice (912 nt, *p* < 0.0001) (Figure 2c). Additionally, the average exon number of mRNAs in wild rice were higher than in cultivated rice (*O. rufipogon* 5.85 vs. *japonica* rice 4.55, *p* < 0.0001; *O. rufipogon* 5.85 vs. *indica* rice 4.18, *p* < 0.0001) (Figure 2d), which was also distinct from the tendencies of lncRNAs. These observations suggested that the domestication process was accompanied by the increasing length and exon number of lncRNAs.

**Figure 3:**
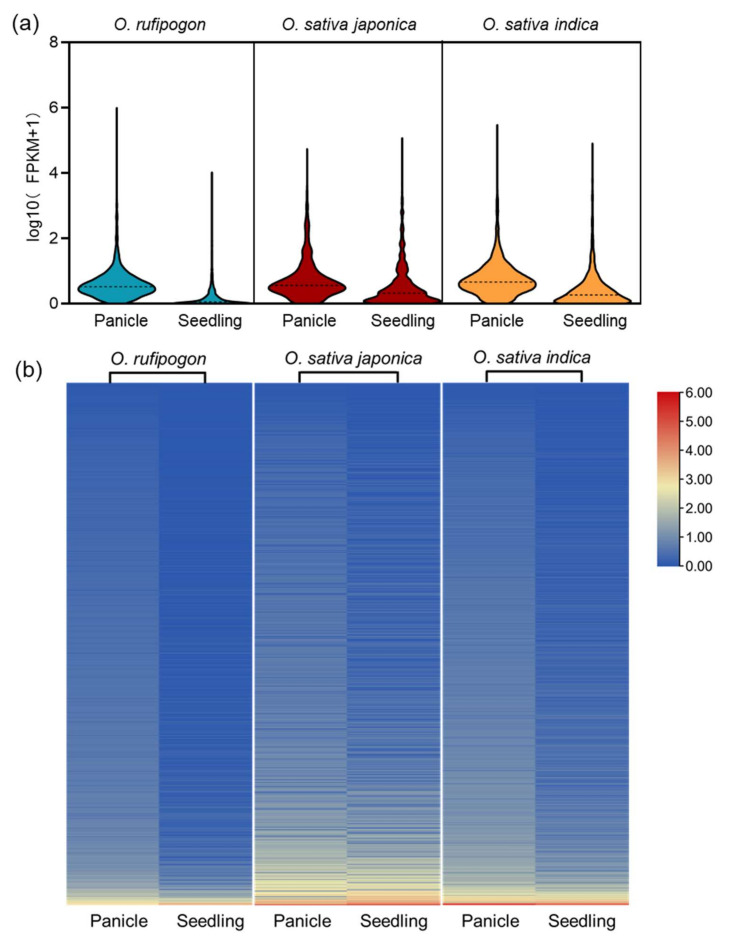
Expression analysis of lncRNAs from wild and cultivated rice. (**a**) The FPKM distribution of lncRNAs in panicle and seedling of wild and cultivated rice. (**b**) Heatmap of differentially expressed lncRNAs in panicle and seedling of wild and cultivated rice. All expression levels are normalized and displayed as log10(FPKM+1). The color from blue to red represents lowest to highest lncRNA expression.

It is reported that a large number of lncRNAs are specifically expressed and involved in the sexual reproduction of rice. We next investigated the expression of lncRNAs in wild and cultivated rice. The results showed that lncRNAs were significantly higher expressed in panicles than in seedlings all through the three *Oryza* genomes. Statistical analysis suggested that the median log10(FPKM+1) value was 0.52 in panicles versus 0.06 in seedlings in wild rice (*p* < 0.0001), while the median values were 0.62 in panicles versus 0.44 in seedlings in *japonica* rice (*p* < 0.0001) and 0.66 in panicles versus 0.26 in seedlings in *indica* rice (*p* < 0.0001) (Figure 3a). A heatmap based on the value of log10(FPKM+1) was also generated to depict the difference in expression level among samples in each lncRNA, from which we could more clearly understand the generally higher expression of lncRNAs in panicles than in seedlings (Figure 3b). Among the 8528 lncRNAs identified in this study, we found a large proportion of lncRNAs were differentially expressed in panicles and seedlings (fold change > 2, and FDR < 0.05) (Supplementary Tables S1–S3), including 3040 out of 4072 lncRNAs (74.66%) in wild rice, 1104 out of 2091 lncRNAs (52.80%) in *japonica* rice, and 1659 out of 2365 lncRNAs (70.15%) in *indica* rice. To confirm the reliability of our datasets for lncRNA expression profiles, we experimentally validated the expression of twelve lncRNAs from wild and cultivated rice with different expression patterns. The results of qPCR analysis revealed that the expression of lncRNAs were consistent with those obtained from lncRNA sequencing (Supplementary Figure S2).

### 2.3. Conservation Analysis of lncRNAs That may be Associated with Rice Domestication

It has been well-characterized that lncRNAs are highly diverged at the nucleotide level among plant species but have high sequence conservation at the intra-species and sub-species levels [22]. We want to know whether lncRNA sequences are conserved or not between rice and its wild ancestor. After the sequence alignment of these 8528 lncRNAs identified in our datasets, we screened only 628 out of 4072 (15.42%) wild rice-expressed lncRNAs with homologous regions with 272 out of 2091 (13.01%) lncRNAs from *japonica* and 246 out of 2365 (10.40%) lncRNAs from *indica*, showing that only a few lncRNAs were sequence-conserved between wild and cultivated rice (Supplementary Table S4).

**Figure 4:**
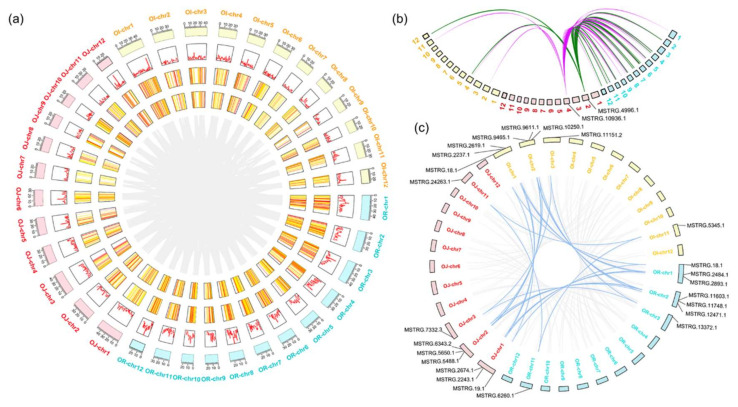
Circos plots of sequence conserved and positionally conserved lncRNAs that may be associated with rice domestication. (**a**) Distribution of sequence-conserved lncRNAs between *O. rufipogon*, *japonica* and *indica*. The 12 chromosomes in each set are shown in the outermost circle. Red lines in the second circle indicate the density of lncRNAs detected in this study. Heatmaps in the third and fourth circles represent the expression level of sequence-conserved lncRNAs in panicles and seedlings, respectively. The internal links in gray connect lncRNAs that are sequence-conserved between *O. rufipogon*, japonica and indica. (**b**) Two examples showed multiple lncRNAs in wild rice are sequence-conserved with rare lncRNAs in cultivated rice. Purple lines indicate 41 lncRNAs in *O. rufipogon* are homologous with 1 and 3 lncRNAs in *japonica* and *indica*, respectively. Green lines indicate 21 lncRNAs in *O. rufipogon* are homologous with 1 and 2 lncRNAs in *japonica* and *indica*, respectively. (**c**) Circos representation of positionally conserved lncRNAs between *O. rufipogon*, *japonica* and, *indica*. Links depicted conserved synteny of lncRNAs between at least two genomes, with blue lines highlighting the eight positionally conserved lncRNA-matches between *O. rufipogon*, *japonica*, and *indica*.

The numbers of conserved lncRNAs with homologous sequences in wild and cultivated rice are not identical, implying that duplication or deletion of lncRNA copies occurred during rice domestication. We then performed an in-depth analysis of these sequence-conserved lncRNAs and a total of 633 matches were constructed among the 628/272/246 lncRNAs in wild/*japonica*/*indica* rice (Figure 4a and Supplementary Table S4). We found multiple lncRNAs in wild rice are homologous to a few of lncRNAs in cultivated rice (Figure 4a,b). MSTRG.10936.1 and MSTRG.4996.1 from *O. sativa japonica* are two examples that are sequence-conserved with 2 and 3 lncRNAs in *indica* but with 41 and 21 lncRNAs in *O. rufipogon*, respectively (Figure 4b). Sequence analyses of these two multiple-matched lncRNAs revealed that they were highly identical to two transposable elements, i.e., terminal-repeat retrotransposon in miniature ZO3 (GenBank accession: EF555578.1, identity: 96.50%) and transposon CACTG element RIM2-M337 (GenBank accession: BK000948.1, identity: 96.39%). These TE-associated lncRNAs are widely expressed in the wild rice, but only a few of them are detected in the cultivated rice, suggesting most of these TE-associated lncRNAs are silenced or excluded during the domestication of rice.

Next, we investigated the gene synteny information to assess the orthologous relationships of lncRNAs (Figure 4c and Supplementary Table S5). By analyzing the collinear segments of lncRNAs, we identified 78 lncRNA-matches comprised of 69 wild rice-derived lncRNAs and 55 japonica rice-derived lncRNAs. In the wild-*indica* rice group, 123 lncRNA-matches comprised of 105 wild rice-derived lncRNAs and 111 *indica* rice-derived lncRNAs were also determined. Only 60 lncRNA-matches comprised of 47 *japonica* rice-derived lncRNAs and 52 *indica* rice-derived lncRNAs showed positional-conservation in the *japonica-indica* rice group. A comprehensive comparison of lncRNA synteny between wild and cultivated rice suggested eight lncRNA-matches were positionally conserved in all the three *Oryza* genomes, indicating potential conserved roles of these lncRNAs during rice domestication (Figure 4c). Additionally, these results also showed that the conserved lncRNA synteny in wild-*indica* rice was stronger than in wild-*japonica* rice, providing evidence from the noncoding RNA perspective to support the latest hypothesis of *indica* rice originated from cross-hybridized ancient *japonica* cultivars and wild rice [9].

### 2.4. LncRNAs Are Potential Targets for the Selection of Yield-Related Traits during Domestication

**Figure 5:**
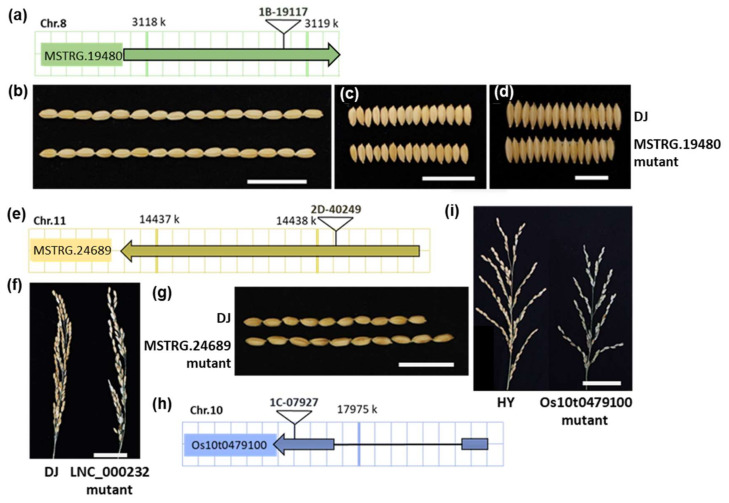
Phenotype analysis of the T-DNA insertion mutants of three conserved lncRNAs in rice domestication. (**a**–**d**) The diagram of T-DNA insertion site in MSTRG.19480 (**a**) and grain size analysis for this mutant and its wild-type Dongjin, the scale bar for grain length (**b**), grain width (**c**) or grain thickness (**d**) is 2 cm. (**e**–**g**) The diagram of T-DNA insertion site in MSTRG.24689. (**e**) and phenotype analysis for this mutant and its wild-type Dongjin. The scale bar is 5 cm for panicle (**f**) and 2 cm for grain length (**g**). (**h**,**i**) The diagram of T-DNA insertion site in Os10t0479100 (**h**) and phenotype analysis for this mutant and its wild-type Hwayoung. The scale bar for panicle (**i**) is 5 cm.

LncRNAs have been identified to be important in plant reproductive development and stress responses [19,29]. To investigate whether the conserved rice lncRNAs during domestication would contribute to agronomic traits, we collected mutants of several conserved lncRNAs to detect their phenotypes. We retrieved the worldwide main rice mutant databases, including Rice Mutant Database [30] and POSTECH [31], and identified three rice mutant lines with T-DNA insertion mutations in three conserved lncRNAs MSTRG.19480, MSTRG.24689 and Os10t0479100 (Figure 5a,e,h). After the screening of homozygous lines of these mutants, we investigated the agronomic traits such as plant architecture, grain morphology, and resistance to *Xanthomonas oryzae* pv. *Oryzae* (*Xoo*) and *Xanthomonas oryzae* pv. *oryzicola* (*Xoc*) pathogens. The results showed that the yield-related traits like panicle architecture, seed-setting rate, grain weight, and size, as well as the performance of these mutants in bacterial resistance, were significantly affected by the expression of conserved lncRNAs in domestication (Figure 5, Table 2 and Supplementary Figure S3).

**Figure 6:**
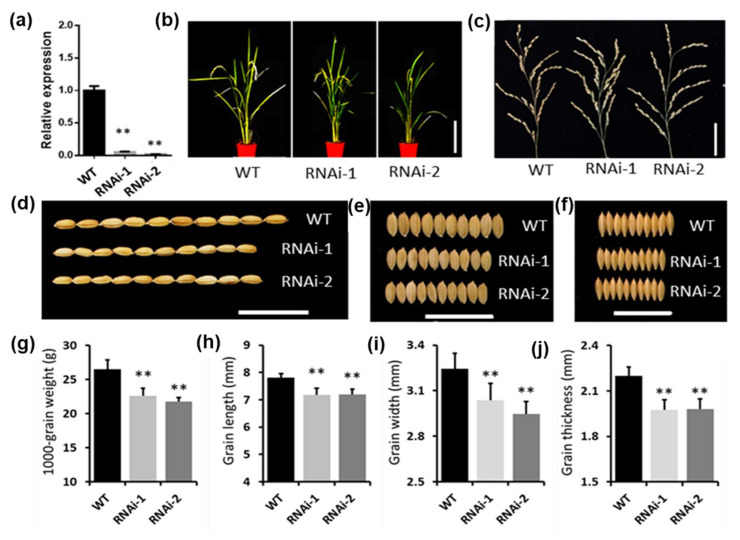
Phenotype analysis of RNAi lines of MSTRG.19480. (**a**) Expression level of MSTRG.19480 in RNAi lines. (**b**–**f**) Phenotype of MSTRG.19480 RNAi lines and wild-type (WT) plants, plant height (scale bar = 20 cm) (**b**), panicles (scale bar = 5 cm) (**c**), grain length (scale bar = 1 cm) (**d**), grain width (scale bar = 1 cm) (**e**), grain thickness (scale bar = 1 cm) (**f**). (**g**–**j**) Statistical analysis of grain weight (**g**), grain length (**h**), grain width (**i**) and grain thickness (**j**) in MSTRG.19480 RNAi lines and WT. Asterisks indicate statistically significant differences compared with WT by Student’s *t* test (** *p* < 0.01; *n* = 50).

MSTRG.19480 locates at 3,117,854 bp to 3,119,120 bp of Chromosome 8 and transcribes a single-exon lncRNA of 1267 nt in length. Its homologous MSTRG.27043 in *O. rufipogon* also transcribes a single-exon lncRNAs of 1187 in length, which could be perfectly matched (percent identity = 100%) to MSTRG.19480. The only difference between these two lncRNAs is that MSTRG.19480 has 5 and 75 additional nucleotides in its 5′ and 3′ ends, respectively (Supplementary Figure S4). To further confirm that conserved lncRNAs are associated with domestication-related traits, we chose MSTRG.19480 for further investigation. We generated transgenic plants of MSTRG.19480 using the RNA interfering technology. The suppressed expression of MSTRG.19480 were verified in RNAi-1 and RNAi-2, two of the 20 transgenic lines (Figure 6a). To investigate the performance of these RNAi lines in domestication-related traits, we cultivated these plants together with the wild-type Nippobare. When compared with the wild-type plants, knockdown of MSTRG.19480 resulted in a decrease in plant height, grain length, grain width, and grain thickness (Figure 6b–j), showing similar phenotypes with its T-DNA insertion mutant (Figure 5b–d). The above observations suggested that the direct target selection on lncRNAs during domestication may contribute to the manipulation of agronomic traits in rice plants.

## 3. Discussion

Crop domestication is generally known as a model system of plant evolution. During this process, human selection drives crop evolution and gives rise to a number of desirable agronomic traits [32,33,34,35]. In the *Oryza* genus, the combination of pan-genome analyses and functional studies has facilitated the identification of many protein-coding genes that are responsible for marked morphological changes, including grain size, flowering, panicle development, plant architecture, and shattering. Recent progress has also revealed that noncoding RNAs widely exist and are essential in regulating plant growth and development [18]. The genome-wide scanning of noncoding RNAs, such as microRNAs and phasiRNAs has been identified not only in cultivated rice but also in wild rice [15,36,37,38,39]. Much of the recent progress indicates that lncRNAs play important roles in plant development, however, limited data are available for their regulation in crop domestication. In this present study, we focused on the phenotypic variations of two cultivated rice varieties, *O. sativa* ssp. *japonica*, and *O. sativa* ssp. *indica*, and their wild progenitor *O. rufipogon*. Morphological and statistical analyses suggested that yield-related traits, such as seed-setting rate, grain size, and grain weight, are significantly increased in cultivated rice than in wild rice. To answer whether lncRNAs are also associated with domestication, we then collected samples of these three *Oryza* species and performed a genome-wide analysis to identify lncRNAs. Comparative analysis and functional study demonstrated that many conserved lncRNAs are associated with agronomic traits in rice. Our data further complemented the context of rice lncRNAs and suggested a potential role of lncRNAs for rice breeding.

Although both *japonica* rice and *indica* rice were developed from wild rice, they experienced separate domestication process from different *O. rufipogon* populations [9]. *Japonica* rice was first domesticated in southern China from one wild rice ecotype, while *indica* rice was later derived from cross-hybridized ancient *japonica* cultivars and other ecotypes of wild rice [9]. A recent study reported 3363 lncRNAs expressed in the panicle of *O. rufipogon* and *japonica* rice [21]. Nevertheless, the characteristics of lncRNAs among *indica* rice, *japonica* rice, and wild rice still need to be elucidated. We subsequently found in this study that the percentage of short and single-exon lncRNAs in wild rice was higher than in cultivated rice, showing a tendency distinct from that of mRNA. We also evidenced higher expression of lncRNAs in panicles all over the three datasets. As the changes of lncRNA length, exon numbers and expression patterns were accompanied by the rice domestication process, we hypothesized that lncRNAs might also be selected during domestication and might have crucial biological roles in regulating rice traits. Based on our following analysis of several lncRNA mutants, we have finally revealed the regulatory role of lncRNAs for domestication traits, suggesting promising prospects for the application of lncRNAs for crop improvement.

We identified 628 lncRNAs in wild rice that are homologous to 272 lncRNAs from *japonica* rice and 246 lncRNAs from *indica* rice. Further analysis showed multiple lncRNAs in wild rice are homologous to a few lncRNAs in cultivated rice, and sequence alignment of two lncRNA examples suggested they are highly identical to two transposable elements. There have been a number of longitudinal studies involving the expression of TE-associated lncRNAs during the domestication of plants. Some TE-associated lncRNAs are proved to be related to stress responses [40,41,42]. For example, the Arabidopsis expressed TE-lncRNA11195 is activated after abiotic stresses or ABA treatment. Genetic evidence revealed that disruption in this lncRNA could increase plant resistance to abscisic acid in root elongation and shoot fresh weight [41]. Our findings of decreasing the number of TE-associated lncRNAs during domestication raised the possibility that unexpected expression of TE-associated lncRNAs would be harmful for plants to conquer biotic or abiotic stress.

Breeding of domesticated traits such as higher productivity has led to reduced genetic diversity and enriched domestication-related genes among different rice accessions [42,43]. The proper expression of conserved lncRNAs is essential for rice. We collected the T-DNA insertion mutants of three sequence-conserved lncRNAs during domestication and found abnormal performance in yield-related traits such as panicle architecture, seed-setting rate, grain weight, and grain size, illustrating that lncRNAs are potential targets for the selection of yield-related traits during domestication. We further construct the RNAi lines of one conserved lncRNA, MSTRG.19480. Phenotypic analysis also confirmed its role in regulating seed size. These results indicated that lncRNAs might also be selected for maintaining or improving the agronomic traits in grass. Although we demonstrated the involvement of MSTRG.19480 in regulating yield-related traits, additional studies are needed to reveal the exact mechanism by which MSTRG.19480 modulates these traits.

## 4. Materials and Methods

### 4.1. Plant Materials and Growth Conditions

The common wild rice (*Oryza rufipogon,* collected from Hainan province), Nipponbare (*O. sativa* ssp. *japonica*) and 93-11 (*O. sativa* ssp. *indica*) were used in this study for sampling and RNA sequencing. Their Seeds were sterilized and then grown on MS medium in the growth chamber at 28 °C with a photoperiod of 10 h light and 14 h dark. The 3-week-old seedlings were then transplanted to a paddy field, and plants were maintained under routine management practices of water and soil nutrition during the rice-growing season of Guangzhou, China. The natural temperature of the growing season in Guangzhou ranged from 23.8–35.2 °C, and the day length ranged from 12.0 to 13.5 h. A total of thirty 2-week-old seedlings and thirty pre-emergence panicles for each *Oryza* species were collected for the following analysis. The rice T-DNA insertion mutants of conserved lncRNAs were collected from POSTECH [31]. The mutant lines were sown together with their wild-type Dongjin or Hwayoung.

### 4.2. RNA Extraction, Library Construction and Sequencing

Total RNA was isolated with the Plant Total RNA Extraction Kit (Magen, Guangzhou, China) from each sample according to the manufacturer’s instructions, followed by digestion with DNase I (Takara, Dalian, Beijing, China). A total amount of 3 μg RNA per sample was used as input material for the RNA sample preparations. The ribosomal RNA was then removed by Epicentre Ribo-zero™ rRNA Removal Kit (Epicentre, Madison, WI, USA), and rRNA free residue was cleaned up by ethanol precipitation. Next, sequencing libraries were constructed using the rRNA-depleted RNA by NEBNext^®^ Ultra™ Directional RNA Library Prep Kit for Illumina^®^ (NEB, Ipswich, MA, USA) following the manufacturer’s recommendations. At last, products were purified (AMPure XP system, Beckman, Brea, CA, USA), and library quality was assessed on the Agilent Bioanalyzer 2100 system. The sequencing was performed on a HiseqTM 2500 machine (Illumina Inc., San Diego, CA, USA) and 125 bp paired-end reads were generated.

### 4.3. LncRNA Identification

Raw reads that contained adapters and low-quality bases with more than 10% of unknown nucleotides or with 50% of low quality (Q-value ≤ 20) bases were further removed by fastp [31] (version 0.18.0). The rRNA mapped reads were removed by using Bowtie2 [31] (version 2.2.8). The resulting clean data were then aligned with the reference genomes using HISAT2 [31] (version 2.1.0) and transcripts were also reconstructed using Stringtie [31] (version 1.3.4). The reference genomes and annotation data were retrieved from the Rice Annotation Project for *japonica* rice (https://rapdb.dna.affrc.go.jp/download/irgsp1.html, accessed on 8 August 2020) and EnsemblPlants for *indica* rice (ASM465v1, https://plants.ensembl.org/Oryza_indica/Info/Index, accessed on 8 August 2020) and wild rice (OR_W1943, https://plants.ensembl.org/Oryza_rufipogon/Info/Index, accessed on 8 August 2020). Transcripts that are longer than 200 nt, FPKM (fragment per kilobase of transcript per million mapped reads) ≥0.1 and CPC (coding potential calculator, version 0.9-r2, http://cpc.cbi.pku.edu.cn, accessed on 30 August 2020) score < 0 [31], CNCI (coding-noncoding-index, version 2) score < 0 [31] are considered as novel lncRNAs identified in this study. All the identified lncRNAs are listed in Supplementary Tables S1–S3.

### 4.4. Expression Analysis

The FPKM value were calculated to quantify the expression of transcripts by using RSEM software [44]. The violin plot and heatmap that showed expression levels of lncRNAs in wild and cultivated rice are generated according to the log10(FPKM+1) value of lncRNAs. The expression levels of twelve lncRNAs were also confirmed by quantitative RT-PCR, performed by using the SYBR Premix Ex TaqTM Kit (Takara, Dalian) following the manufacturer’s instructions. The rice *ACTIN2* gene levels were used for normalization. LncRNAs relative expression levels were analyzed on QuantStudio™ 6 Flex Real-Time PCR System (Applied Biosystems, Foster City, CA, USA).

### 4.5. Conservation Analyses

The conserved regions in wild-*japonica*, wild-*indica*, and *japonica*-*indica* rice pairs were identified by BLAST with sequence alignment. The homologous lncRNA sequence number >100 nt and percentage identity >90% in wild-*japonica*, wild-*indica*, and *japonica*–*indica* were screened, and common homologous sequences appeared in all the three groups were selected as sequence-conserved lncRNAs. The positional conservation of lncRNAs was analyzed using a method described previously [22]. The nearest upstream and downstream protein-coding genes are used to perform pairwise collinearity analyses based on the genomes of wild and cultivated rice. If 4 out of 5 genes are close in their genomic loci, the lncRNA in the region is considered to be positionally conserved lncRNAs.

### 4.6. Vector Construction and Rice Transformation

To generate the RNAi plants for suppressing the expression of MSTRG.19480, a 455 bp DNA fragments of MSTRG.19480 from Nipponbare was amplified by using the primer pairs F: 5′-GTTGTACCTTTGGAAATTCT-3′ and R: 5′-ACTATAATTCATTATGTTGT-3′. The PCR product was subcloned into pRNAi-35S plasmid in both the sense and antisense orientations to generate a stem-loop structure. The resulting plasmid was then transformed into Nipponbare by using *Agrobacterium tumefaciens*-mediated rice transformation. Transgenic lines were screened by 50 mg/L hygromycin B (Biofroxx, Einhausen, Germany) in MS medium.

### 4.7. Plant Inoculations

To detect the resistance of rice to bacterial blight, flag leaves of the mutant lines were inoculated with the *Xoo* by the leaf-clipping method as described [19]. The leaf tip is cut with scissors previously dipped in PXO99A bacterial suspension with an OD600 of 0.2. The blight lesion lengths were measured 14 days after infiltration. As to the resistance of these mutants to bacterial leaf streak disease, the penetration method with a syringe was used to inoculate flag leaves with *Xoc* strain. Lesions were photographed and measured 14 days after inoculation.

### 4.8. Phenotypic Analyses

The statistically significant differences analyses in this study were performed by Student’s *t*-test. For the comparison of grain morphology of wild and cultivated rice, the seeds were hulled before the measurement to eliminating the influence of awn length. For the comparison of grain morphology of lncRNA mutants and their wild-type seeds, the grains with their hull were used. All the experiments were designed with replicates to measure phenotypic variables. The number of observations (*n* values) used to calculate the mean value are marked in the figure legends and table legend as indicated.

## 5. Conclusions

In this study, we found a number of lncRNAs expressed in wild rice were revealed to be shorter in length and have fewer exon numbers when compared with lncRNAs from cultivated rice. Comparative analysis and the functional study demonstrated that many conserved lncRNAs are associated with yield-related traits in rice. Collectively, our results present the lncRNA characteristics and conservation during rice domestication and demonstrate the potential to improve plant agronomic traits by manipulating the expression of lncRNAs.

## Figures and Tables

**Table 1:** A summary of data from lncRNA sequencing.

Index	*O. rufipogon*		*O. sativa* ssp. *japonica*		*O. sativa* ssp. *indica*	
	Panicle	Seedling	Panicle	Seedling	Panicle	Seedling
Raw reads	82,586,298	90,574,892	88,408,434	97,907,538	93,570,604	104,287,794
Clean reads	82,578,958	90,569,210	88,402,696	97,902,546	93,564,262	104,279,248
Clean bases	12.38 G	13.58 G	13.25 G	14.68 G	14.03 G	15.64 G
GC content	51.09%	46.50%	49.66%	48.10%	49.67%	48.91%
Total mapped reads (%)	62,020,407 (80.10%)	75,227,980 (83.74%)	80,629,086 (92.84%)	91,211,211 (94.06%)	81,866,743 (89.13%)	97,171,107 (95.20%)
Unique mapped reads (%)	61,024,157 (78.82%)	73,364,876 (81.67%)	67,553,409 (77.79%)	60,706,447 (62.60%)	74,933,904 (81.59%)	78,992,468 (77.39%)
Multiple mapped reads (%)	996,250 (1.29%)	1,863,104 (2.07%)	13,075,677 (15.06%)	30,504,764 (31.46%)	6,932,839 (7.55%)	18,178,639 (17.81%)
Reads map to ‘exon’ (%)	32,898,026 (53.04%)	30,524,827 (40.58%)	34,735,578 (43.08%)	17,715,152 (19.42%)	31,530,937 (38.51%)	30,706,672 (31.60%)
Reads map to ‘intron’ (%)	8,676,838 (13.99%)	6149,971 (8.18%)	5,247,590 (6.51%)	2,080,358 (2.28%)	6,717,453 (8.21%)	2,175,025 (2.24%)
Reads map to ‘intergenic’ (%)	20,445,543 (32.97%)	38,553,182 (51.25%)	40,645,918 (50.41%)	71,415,701 (78.30%)	43,618,353 (53.28%)	64,289,410 (66.16%)

**Table 2:** Agronomic traits of mutants of three conserved lncRNAs during domestication.

lncRNA ID	WT-Dongjin	MSTRG.19480	MSTRG.24689	WT-Hwayoung	Os10t0479100
Mutant ID (wild-type)	Dongjin (DJ)	1B-19117 (DJ)	2D-40249 (DJ)	Hwayoung (HY)	1C-07927 (HY)
Plant height (cm)	64.38 ± 3.56	51.38 ± 3.87 (**↓)	59.62 ± 4.45 (**↓)	64.50 ± 4.54	56.92 ± 3.04 (**↓)
Panicle length (cm)	16.80 ± 0.56	16.62 ± 2.04 (n.s.)	15.76 ± 1.14 (**↓)	16.35 ± 1.44	15.73 ± 1.32 (n.s.)
Number of primary branches	6.57 ± 0.82	6.06 ± 1.57 (n.s.)	6.67 ± 0.85	5.82 ± 0.57	6.57 ± 0.82 (n.s.)
Seed-setting rate (%)	90.52 ± 5.05	90.61 ± 5.01 (n.s.)	67.53 ± 5.08 (**↓)	95.00 ± 4.57	11.01 ± 3.93 (**↓)
1000-grain weight (g)	25.56 ± 0.56	20.66 ± 2.93 (**↓)	26.40 ± 1.34 (*↑)	24.75 ± 1.21	24.80 ± 0.64 (n.s.)
Grain length (mm)	7.64 ± 0.33	6.93 ± 0.27 (**↓)	8.28 ± 0.26 (**↑)	7.94 ± 0.29	8.02 ± 0.25 (n.s.)
Grain width (mm)	3.49 ± 0.14	2.72 ± 0.17 (**↓)	3.43 ± 0.14 (n.s.)	3.56 ± 0.12	3.61 ± 0.16 (n.s.)
Grain thickness (mm)	2.33 ± 0.09	2.08 ± 0.19 (**↓)	2.31 ± 0.06 (n.s.)	2.18 ± 0.08	2.14 ± 0.08 (n.s.)
Lesion length of *Xoo* infection (cm)	6.56 ± 1.99	4.73 ± 1.68 (**↓)	5.71 ± 2.97 (n.s.)	5.31 ± 2.29	9.10 ± 2.99 (**↑)
Lesion length of *Xoc* infection (cm)	2.11 ± 0.76	2.30 ± 0.22 (n.s.)	0.47 ± 0.18 (**↓)	1.15 ± 0.76	2.19 ± 0.83 (**↑)

Asterisks indicate statistically significant differences compared with wild-type by Student’s *t*-test (* *p* < 0.05, **p < 0.01), n.s., not significant. Arrows indicate up- or down-regulation when compared with the wild-type plant Dongjin or Hwayoung. The sample numbers are *n* > 10 for plant and panicle architectures, *n* > 30 for grain traits, and *n* > 8 for *Xanthomonas oryzae* pv. *Oryzae* (*Xoo*) and *Xanthomonas oryzae* pv. *oryzicola* (*Xoc*) infection.

## Data Availability

The datasets used in this study are included in the article and its additional files. The strand-specific transcriptome sequencing of *Oryza* samples generated in this study was submitted to NCBI Sequence Read Archive database with the BioProject accession of PRJNA694829.

## Supplementary Materials

The following are available online at https://www.mdpi.com/article/10.3390/ijms22094742/s1, Figure S1: Validation of lncRNA expression profiles in RNA-seq by quantitative real-time PCR, Figure S2: Comparison of resistance to *Xoo* (a) and *Xoc* (b) between wild type and T-DNA insertion mutants of three conserved lncRNAs in rice domestication, Figure S3: Comparison of resistance to Xoo (a) and Xoc (b) between wild type and T-DNA insertion mutants of three conserved lncRNAs in rice domestication, Figure S4: Sequence alignment of MSTRG.19480 in japonica rice and MSTRG.27043 in O. rufipogon, Table S1. Genomic loci and expression pattern of lncRNAs identified from *Oryza rufipogon,* Table S2. Genomic loci and expression pattern of lncRNAs identified from *Oryza sativa* spp. *Japonica*, Table S3. Genomic loci and expression pattern of lncRNAs identified from *Oryza sativa* spp. *Indica,* Table S4. Sequence-conserved lncRNAs between wild and cultivated rice, Table S5. Positionally conserved lncRNAs between wild and cultivated rice.
