# Supplementary material for: Genome-Wide Analysis Identified a Set of Conserved lncRNAs Associated with Domestication-Related Traits in Rice
Source: Int J Mol Sci. 2021 Apr 29;22(9):4742. doi: 10.3390/ijms22094742 (PMC8124408; doi:10.3390/ijms22094742)
Supplement: Supplementary file 1 [file ijms-22-04742-s001.zip › Supplementary figures.pdf]

International Journal of
Molecular Sciences

MDPI

Supplementary figures and legends

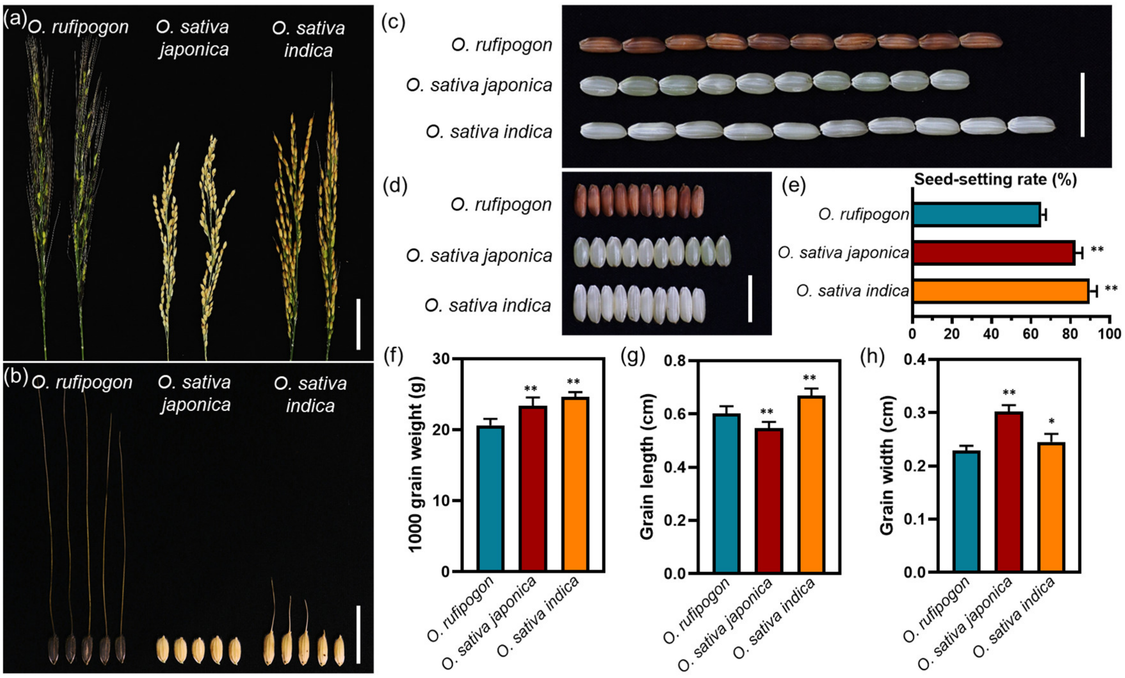

Supplementary Fig. S1. Phenotypic comparisons between wild rice and two cultivated rice varieties. The common wild rice (*Oryza rufipogon*, collected from Hainan province), Nipponbare (*O. sativa* ssp. *japonica*) and 93-11 (*O. sativa* ssp. *indica*) was used in this study. **(a)** Panicle architecture. The scale bar is 5 cm. **(b)** Grain morphology. The scale bar is 2 cm. **(c-d)** Grain length (c) and width (d). The scale bar is 1 cm. **(e-h)** Data analysis of seed-setting rate (e), 1000-grain weight (f), grain length (g) and grain width (h). Data are presented as means ± SD. Asterisks indicate statistically significant differences compared with the *Oryza rufipogon*, as determined using Student's *t* test (*P < 0.05; **P < 0.01; n = 30).

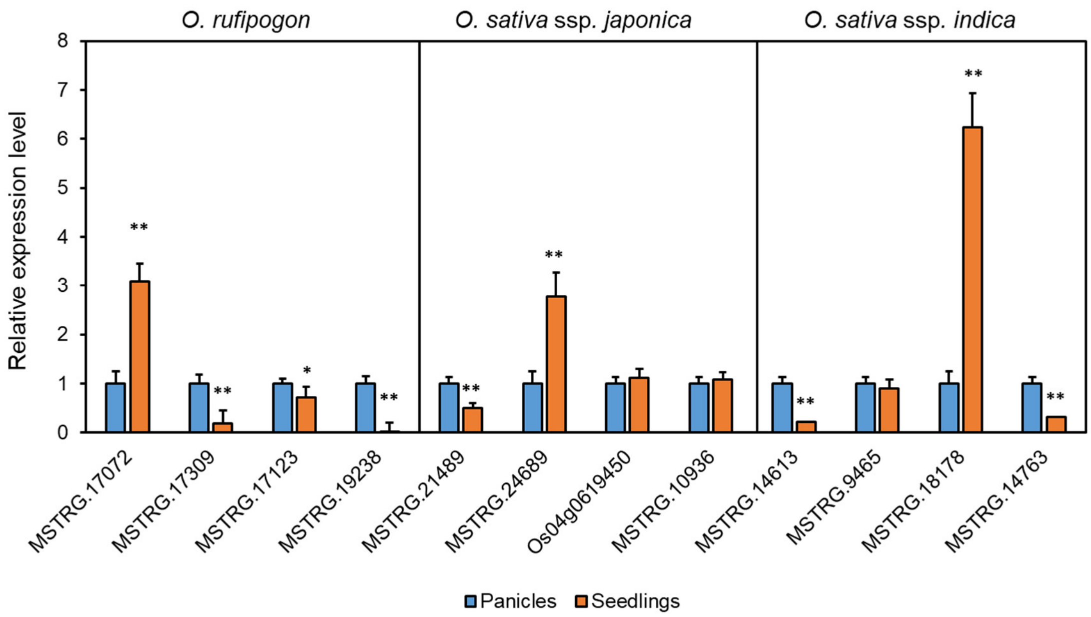

Supplementary Fig. S2. Validation of lncRNA expression profiles in RNA-seq by quantitative real-time PCR. Rice gene *Actin* 2 was used as an endogenous control. Expression levels of panicles were set to 1. The data are expressed as the mean ± SD of three replicates. Asterisks indicate statistically significant differences compared with wild rice by Student's *t* test (*p<0.05, **p< 0.01).

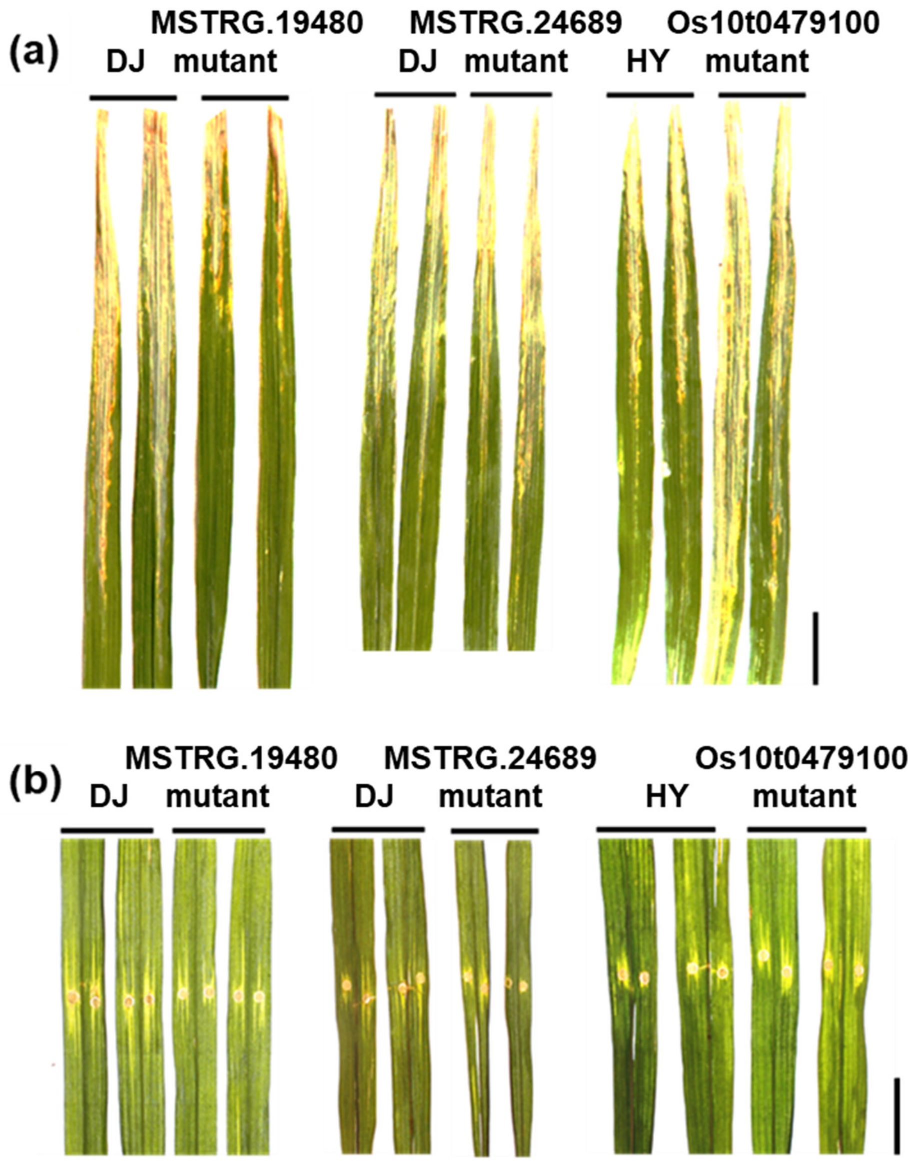

Supplementary Fig. S3. Comparison of resistance to *Xoo* (a) and *Xoc* (b) between wild type and T-DNA insertion mutants of three conserved lncRNAs in rice domestication. Scale bar =5 cm.

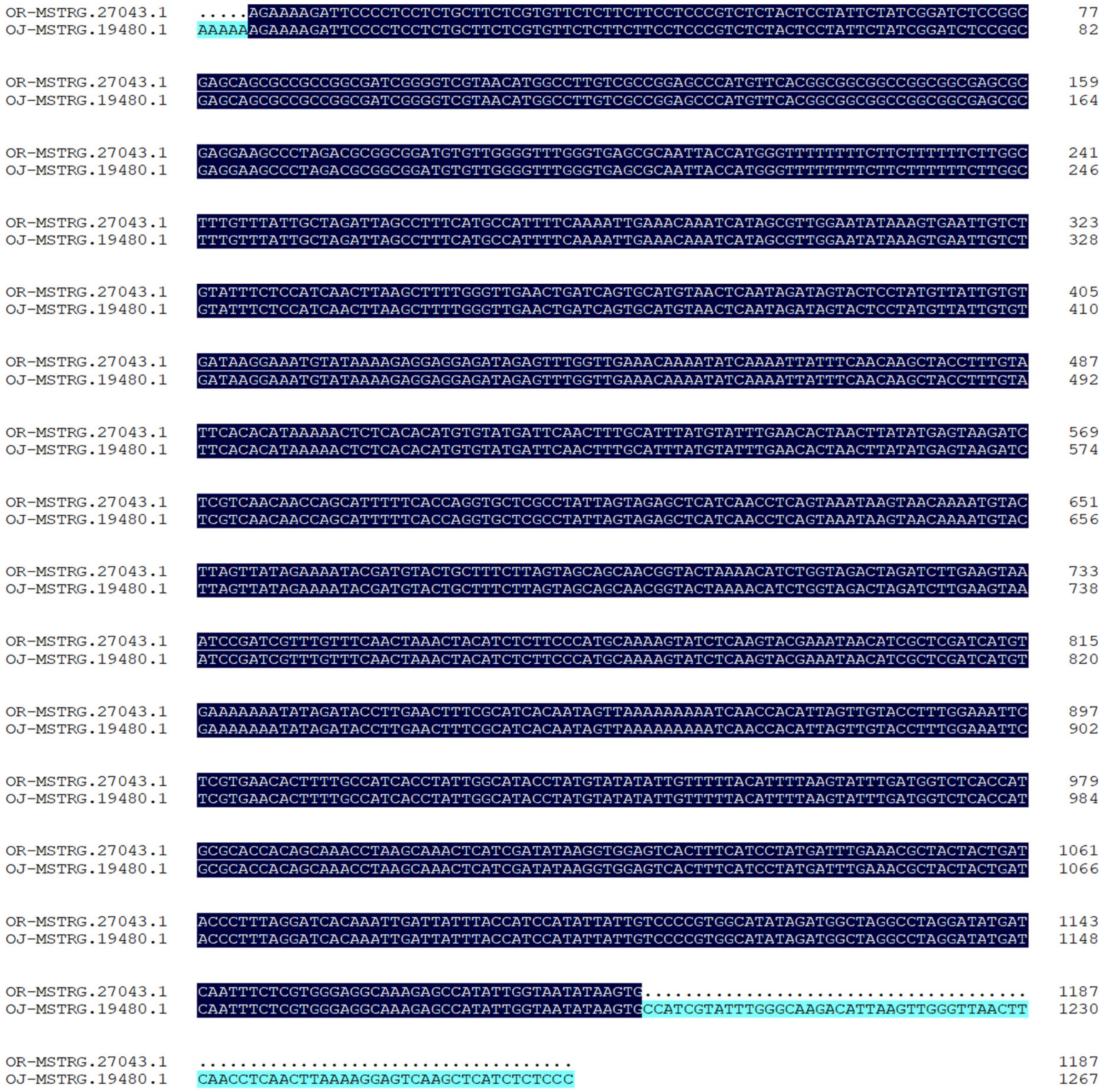

Supplementary Fig. S4. Sequence alignment of MSTRG.19480 in *japonica* rice and MSTRG.27043 in *O. rufipogon*.
